# Agency and resilience among young persons in Sweden: Patterns of interrelated resources within the WHO Adolescent Well-being Framework

**DOI:** 10.1371/journal.pone.0360518

**Published:** 2026-10-30

**Authors:** Johanna Bergman, Albert Westergren, Åsa Bringsén, Petra Nilsson Lindström, Maria Haak

**Affiliations:** 1 Faculty of Health Sciences, Kristianstad University, Kristianstad, Sweden; 2 Department for Research, Development and Education, Management for Psychiatry and Habilitation, Region Skåne, Lund, Sweden; National Taichung University of Science and Technology, TAIWAN

## Abstract

The WHO Adolescent Well-being Framework identifies agency and resilience as a central domain of adolescent well-being, yet its expression from a health promotion perspective remains insufficiently understood. Although previous research has often focused on specific behaviours or isolated factors, less attention has been given to how interconnected personal and relational resources coexist as patterns reflecting the WHO domain of agency and resilience. This study explored such patterns among young Swedish persons aged 15–18 years and examined gender differences across these patterns. A cross-sectional survey was conducted among 419 students in Swedish upper secondary schools. Agency and resilience were operationalised through measures of salutogenic health, positive youth development (PYD), playfulness, goal orientation, and physical activity. A person-oriented cluster analysis was used to identify patterns of interconnected resources, and gender differences between patterns. Three distinct resource patterns were identified: a low-resource group (26%), a high-resource group (28%), and an asymmetric-resource group (47%), characterised by relatively favourable intrapersonal resources alongside lower relational PYD resources. Girls were significantly overrepresented in both the low-resource and asymmetric-resource groups. Together, these findings suggest that young persons with seemingly favourable individual resources may nevertheless exhibit underlying vulnerabilities when resources are considered as interconnected patterns rather than isolated characteristics. This perspective contributes to the empirical understanding of the WHO domain of agency and resilience and may inform person-centred, relational approaches to promoting young persons’ well-being.

## Background

Navigating the health and well-being landscape of young persons is challenging, yet it remains a vital topic due to its importance for both individuals and society. The developmental years are a critical period of life during which behaviours and factors that contribute to lifelong well-being can be learned and established [1,2]. However, self-rated health has declined among younger persons since 2018 and gender differences in health tend to emerge already during adolescence [3]. Girls generally report lower self-rated health and lower life satisfaction, as well as poorer mental well-being than boys, and these differences tend to widen throughout the adolescent years [3]. As researcher emphasise the importance of improving well-being during the developmental years, a specific definition addressing well-being for persons aged 10–19 has been proposed by World Health Organisation (WHO) for guiding international policy development to promote health and health equality:”*Adolescents have the support, confidence, and resources to thrive in contexts of secure and healthy relationships, realizing their full potential and rights”* [4].

The definition underscores both intra- and interpersonal dimensions of well-being in the early years of life, emphasising young persons’ opportunities to realise their full potential and rights through the contexts and relationships that shape their development. These dimensions are further described by Ross et al. [1] as five domains of the Adolescent Well-being Framework: Agency and Resilience, Connectedness, Family Dynamics, Health and Nutrition, Learning Competence, and Safety. Among these domains, the present study specifically focuses on agency and resilience. The agency and resilience domain concerns young persons’ capacity to act intentionally, navigate challenges, and move towards meaningful goals and future possibilities. In this context, agency refers to the perceived capacity to influence one’s life circumstances and act towards desired goals, while resilience concerns adaptive processes that support positive development through changing life conditions and challenges. Both processes develop through the interaction between individual, relational, and contextual resources.

In a recent systematic review, Banati et al. [2] examined experiences and exposures among persons aged 10–19 in relation to interventions targeting the five well-being domains informed by the framework proposed by Ross et al. [1]. The review found that the health and nutrition domain, encompassing physical health, nutritional status, diet, and physical activity was the most frequently investigated. Nonetheless, evidence from this domain demonstrated weak predictive value for key later-life outcomes such as health, exposure to violence, education, and employment. In contrast, the agency and resilience domain, which includes studies on identity formation and the capacity to act intentionally when facing challenges (e.g., goal setting, autonomy, adaptability, sense of purpose, and optimism), was the least studied but yielded the strongest and most consistent predictive associations with later outcomes, particularly health, employability, and safety.

Despite its importance, the agency and resilience domain remains relatively unexplored in younger age groups in relation to health promotion. One possible explanation is that these concepts are commonly studied within psychology in direct relation to stress, trauma, or severe adversity, rather than as everyday developmental and health-promoting processes [5]. In the present study, these processes are approached from a health promotion perspective and explored through psychological, relational, motivational, and behavioural resources relevant to young persons’ everyday lives.

To operationalise these resources, six interrelated constructs linked to the agency and resilience domain of the Adolescent Well-being Framework are introduced and examined: salutogenic health, positive youth development (PYD), playfulness, task and ego orientation, and physical activity. Together, these constructs represent complementary dimensions of resources young persons may use navigate challenges, engage with their surroundings, pursue goals, and experience well-being in everyday life.

The salutogenic orientation of health appreciates that life is dynamic and commonly uses the metaphor of “river of life”. This metaphor acknowledges that by being alive, we are all in the river, hence no one is safe at the shore and can be “protected” from the potential forces of the river [5,6]. Instead, persons need to be taught how to swim and navigate the dangerous parts of the river, highlighting that salutogenic health also involves a sense of agency, that is the capacity to act, make choices, and influence one’s own path. From the perspective of the Adolescent Well-being Framework, salutogenic health may therefore reflect how young persons draw upon personal and contextual resources to navigate everyday life challenges. Furthermore, the orientation explains that suffering can be a source for learning and events in our lives can influence and form persons’ health practices [7,8]. Salutogenic health orientation views health as an overall subjective positive experience in relation to oneself and emphasises that health is continuously formed within the contexts of everyday life [6,7]). From a salutogenic orientation, subjective experience of health can therefore be explored through indicators related to physical, psychological, and social well-being [9].

The framework of PYD shares both the salutogenic strength-based orientation and the relational aspect of the Adolescent Well-being Framework focusing on creating conditions that enable young persons to realise their potential [10–13]. Rather than focusing solely on creating change within the individual, the PYD framework emphasises collaboration with young persons across different settings and acknowledges these relationships as mutually beneficial [14]. This relational perspective is particularly relevant in relation to resilience, as previous research has highlighted the importance of considering resilience as a collective and contextual process rather than solely an individual characteristic [15,16]. Studies within the PYD framework has indicated that higher levels of experienced PYD have been associated with less risky behaviours, improved school satisfaction, and sense of thriving in various cultures [12,13,17–19]. Research within the field of PYD has also demonstrated gender and cultural differences [20], where aspects relating to empathy, caring for others, and moral values, tend to receive higher ratings among girls, while aspects related to confidence t and competence are more strongly endorsed by boys [20,21].

While PYD highlights relational resources related to agency and resilience, playfulness draws attention to how persons engage with and frame their everyday experiences. Playfulness concerns the capacity to approach situations in ways that are enjoyable, imaginative, or thought-provoking [22], and has been linked to well-being through processes related to reframing situations for learning, self-improvement, and adaptation regardless of adversity [23,24]. Playfulness may function as a cognitive and emotional resource that supports flexible engagement with challenges and everyday situations. It may also encourage exploratory and intrinsically motivated ways of interacting with the surrounding world [22]. In a study including persons ranging from the age of 20–79, playfulness was identified as a potential integrative resilience resource that could promote individual flourishing [24]. Consequently, playfulness may also represent an important resource for understanding resilience-related processes in younger age groups. Playfulness can be considered both as a trait and a behavioural attribute, and in this study, it will be considered from a trait perspective [20,25].

Playfulness is arguably linked to intrinsic motivation, which the two-dimensional Goal Achievement Theory Framework describes as mastery or task orientation, and may be viewed as a motivational resource related to agency and resilience [26]. The second dimension, ego orientation, focuses on competition, comparison, and outperforming others, thereby reflecting a different motivational approach to competence and achievement. The Achievement Goal Theory was originally developed in relation to academic achievements but has later been widely applied within sport and physical activity contexts as well [26,27]. The two orientations, although not bipolar, suggest different mechanisms influencing how persons set goals, interpret competence, and engage in physical activity and sport [28,29]. This is relevant as physical activity appears to support positive development during the developmental years through its influence on cognitive and emotional systems and at the same time, it may provide opportunities for social participation, mastery experiences, and experiences of competence [30,31]. Furthermore, Wang et al. [32] found that interventions combining physical activity with psychological training reduced negative thinking among persons aged 15–60. Hence, physical activity may therefore provide additional insights into the resources associated with agency and resilience according to the Adolescent Well-being Framework.

Although agency and resilience have been identified as important predictors of later-life outcomes, the domain remains comparatively underexplored from a health promotion perspective. Existing research has often focused on specific behaviours or isolated factors, whereas less attention has been given to how different psychological, relational, motivational, and behavioural resources may coexist and form distinct patterns within young persons’ lives. Examining how these resources coexist may contribute to a more nuanced understanding of the domain, if agency and resilience emerge through constellations of personal and relational resources rather than isolated factors.

The present study approaches the agency and resilience domain through a set of constructs that have been theoretically linked to young persons’ resources to act intentionally, pursue goals, adapt to challenges, and experience positive development. By exploring salutogenic health, PYD, playfulness, task and ego orientation, and physical activity together, the study seeks to provide a more nuanced understanding of the resources associated with agency and resilience during adolescence. Such knowledge may contribute to both younger persons’ well-being research and health-promoting efforts targeting young persons. Furthermore, gender differences warrant attention, as girls consistently report lower well-being and higher levels of academic stress in national surveys and reports [3,33,34].

The aim of this study is to identify and characterise patterns in how salutogenic health, PYD, playfulness, task and ego orientation, and physical activity co-occur in a sample of Swedish persons aged 15–18 enrolled at upper secondary schools. Furthermore, gender differences will be investigated.

## Method

To capture naturally occurring constellations of resources, and to describe patterns that may characterise distinct expressions of agency and resilience, the study applied cluster analysis. Cluster analysis has been referred to as a person-oriented analytical approach, as it seeks to identify naturally occurring patterns in the sample rather than imposing pre-determined cut-off points or focusing on the variable-based relationships between dependent or independent variables [35]. The study employed a cross-sectional design and was informed by the STROBE checklist [36].

### Setting and sample

The study was conducted within the Swedish upper secondary school system, which includes students aged 15–18. After compulsory schooling, eligible students can choose among 18 national programmes: six higher-education preparatory and twelve vocational tracks. The preparatory programmes focus on academic study and provide eligibility for university admission, whereas the vocational ones prepare students for direct entry into working life, though they can include optional courses granting higher-education eligibility [37]. There were approximately 372 000 students enrolled in upper secondary schools 2024/2025 in Sweden (48.7% girls and 51.3% boys) and girls are more commonly enrolled on higher-education preparatory tracks and boys more commonly enrolled on vocational tracks [37].

A total of 42 upper secondary schools in the southern region of Sweden were informed about the study and invited to participate between April and October 2024. The schools were purposefully selected to ensure representation of both rural and urban contexts as well as spanning across all 18 different national programmes. As cooperation with upper secondary schools was part of the sampling, only persons enrolled within the school system had the opportunity to be part of the study.

The study is part of a larger PhD project where a work group with representatives (both teachers and students) from upper secondary schools have been advised continuously in study design, analysis and communication of results within the project to avoid biases and to bring in different perspectives.

### Instruments

A battery of validated instruments (presented below and in Table 1) was used to operationalise the introduced constructs related to WHO Adolescence Well-being domain of agency and resilience (SHIS, PYD, SMAP, TEOSQ, and physical activity level). The battery also included background variables: gender identification (girl, boy, non-binary, other or do not want to answer), attending school year, and educational programme.

**Table 1:** Rating scales used in the current study.

Instrument/single item	Number of items	Score range	Score interpretation	References
Salutogenic Health Indicator Scale (SHIS)	12	12-72	higher = better	(7)
PYD-Very Short Form (PYD-VSF): Domains	17		higher = better	(35)
Self-worth	10	0-40^a^		(36)
Pro-social	7	0-28^a^		
Short Measure for Adult Playfulness (SMAP)	5	5-25	higher = better	(22, 37)
Task and Ego Orientation in Sport Questionnaire (TEOSQ): Domains	13			(23)
Task	7	1-5	higher = mastery orientated	
Ego	6	1-5	higher = competitive orientated	
Physical activity level	1	1-6	higher = better	(38)

^a^Transformed linear score range

### Salutogenic Health Indicator Scale (SHIS)

Salutogenic health was measured using SHIS. The instrument is a self-reported 12-item questionnaire designed to measure salutogenic and holistic indicators of health [9]. The items ask the respondents to reflect upon how they have felt in the last 4 weeks regarding 12 different salutogenic health indicators. The items are worded in a semantic differential manner where the answers go from most positive to the most negative using a six-point Likert scale. An example of an item is: *“In the last 4 weeks, I have felt alert – I have felt tired, exhausted”.*

The Likert scale is scored from positive (6p) to negative (1p). Summing the twelve items produced a total score ranging from 12 to 72.

SHIS has been validated as a unidimensional and brief assessment tool for saultogenic health for the agegroup 13–15 [38].

### PYD-Very Short Form (PYD-VSF)

PYD-VSF was used to measure the experience of positive youth development. The instrument consists of 17 items and is a short form of the original instrument PYD 5C consisting of 72 items [39]. The instrument is based on the 5C model within PYD framework [10]. The model identifies the five core components that builds up PYD-VSF: *Competence, Confidence, Connection, Character*, and *Caring* [39]. Items related to *Competence* refers to a positive evaluation of one’s performance in specific domains, including social, academic, cognitive, health, and vocational areas. *Confidence* items denote an internal sense of overall self-worth and perceived ability. *Connection* items capture the presence of positive, bidirectional bonds with people and institutions, such as peers, family, school, and community, emphasizing the mutual nature of these relationships. *Character items* relate to respect for societal and cultural norms, adherence to moral standards, a well-developed sense of right and wrong, and integrity. *Caring* items involve both the experience of being cared for and caring for others and is associated with feelings of sympathy and empathy.

A Swedish psychometric study [40] with persons aged 15–18 found support for a unidimensionality and an underlying two-construct structure of the scale was indicated. The two domains differentiated as *self-worth* (Competence, Confidence and Connection) and *pro-social* (Character and Caring) and raw scores were transformed to linear measure where Self-worth score range from 0–40 and Pro-social from 0–28. Analysing the scores using the two underlying but related domains is of theoretical significance, hence, the results of the two domains and their linearised scores will be used in this analysis. All items are rated on a five-point Likert scale ranging from 1 (*Strongly disagree*) to 5 (*Strongly agree*) where higher scores indicate more experienced Self-worth and Pro-social attributes.

### Short Measure for Adult Playfulness (SMAP)

Playfulness was measured using SMAP. The SMAP instrument was developed by Proyer [25]. SMAP is designed to measure playfulness as a trait in adults. It’s a brief, self-reported five-item scale designed to assess the ease of initiation and intensity of playful experiences, as well as the frequency of engagement in playful activities. An example of an item is: *“I easily start playing/being playful”.* The Swedish version used in this study uses a five-point Likert scale [41], but English and German versions have adopted both four- and seven-point Likert scales with good validity [42]. Furthermore, Lau et al. [42] found support for undimensionality, construct validity and high test-retest reliability with both four- and seven-point Likert scales in a population aged 16–36 years. Summation of the SMAP has a score ranging from 5p to 25p, and higher scores means more of the trait playfulness.

### Task and Ego Orientation in Sport Questionnaire (TEOSQ)

TEOSQ was used to measure goal orientation in relation to physical activity and to understand behaviour in relation to physical activity [26]. It is a self-reported instrument developed to assess respondents’ levels of task and ego orientation within sports using two subscales (task and ego). It contains 13 items, seven forming the task orientation subscale, and six forming the ego orientation subscale. Each item begins with the statement *“I feel most successful in physical activity or sport when…”* and an example of an item is: *“I learn a new skill, and it makes me want to practice more”*. Respondents indicate their agreement on a five-point Likert scale from *Strongly disagree (1)* to *Strongly agree* (5). The average score on each subscale is calculated with higher scores indicating higher orientation.

A Mokken Scale Analysis of the TEOSQ based on data from 1452 16-year-old football players in Iceland showed medium scalability for both subscales, indicating primarily suitability for group-level analysis in its current form [43].

### Physical activity level

A single item asking for anticipated physical activity level with a six-point Likert scale (*Never or almost never, about once a month, a few times a month, about once a week, several times a week, daily or almost daily*). The single item is recurrent used in national Swedish surveys for the target group [44,45].

### Data collection

Cognitive interviews were held with fifteen persons aged 15−18 (seven girls, eight boys) from two different upper secondary schools (one rural and one urban) to evaluate the user-friendliness of the battery of questionnaires and estimate time of completion. The persons participating in the cognitive interviews received an information letter stating the ethical approval diary number and that participation was voluntary, with the right to withdraw at any time and no interview were recorded, only notes were taken and average completion time was estimated. Verbal informed consent was obtained and confirmed through participants’ active engagement in the interview. The procedure of the cognitive interviews was approved by the Swedish Ethical Review Authority (Diary number 2024-01088-01).

Following the cognitive interviews the 42 purposefully selected schools were informed about the study and were provided with a digital survey link and a corresponding QR code generated via the platform EvaSys [46]. The survey was open between 17^th^ April 2024 to 1^st^ October 2024. The QR code and link could be distributed through internal school communication channels or by posting printed materials within the school environment. Distribution by the schools and participation was voluntary. Upon accessing the survey, participants were presented with an information letter outlining the purpose of the study, an estimated completion time of 5–10 minutes and contact information for support services in the event of health-related concerns arising from participation. The letter also clarified that submitting the completed questionnaire constituted informed consent, and that participants retained the right to withdraw by exiting the survey at any point before submission. The procedure of informed consent was approved by the Swedish Ethical Review Authority (Diary number 2024-01088-01), and according to the Ethical Review Act section 13–19 [47], guardians’ consent is not required for participants aged 15–18. No personal information was collected; hence, anonymity was maintained during and after the data collection

### Statistical analysis

Descriptive statistics were calculated using SPSS (version 30) and the data was explored for descriptive statistics and normality of the scores was based upon skewness and kurtosis with a criterion of ranging between −2 and 2 [48]. Comparison between genders total scores were performed using independent t-test.

All variables’ scores were then converted to standardised scores (Z-scores) to enable comparison across the different measurement scales in the cluster analysis. Cluster analysis was conducted in SPSS (version 30) to identify naturally occurring patterns of the five constructs based on the instruments *SHIS, PYD-VSF, SMAP, TEOSQ* and *Physical activity level.* First, a hierarchical cluster analysis using Ward’s method (Squared Euclidean distance) was conducted to determine the optimal number of clusters in the sample. Ward’s method was used in the hierarchical procedure to minimize within-cluster variance. Visual inspection of the dendrogram and a marked increase in the agglomeration coefficient guided the decision of number of clusters. Alternative cluster solutions were explored during the analytical process. A four-cluster solution was considered but rejected due to small cluster sizes and limited interpretability, supporting the robustness and interpretability of the final three-cluster solution. Subsequently, a non-hierarchical K-means cluster analysis was performed using the three-cluster solution suggested by the hierarchical analysis derive the final cluster solution.

We applied Z-score thresholds of ±0.2 to interpret the cluster profiles in terms of ‘low,’ ‘medium,’ and ‘high’ levels across the included variables. The threshold was selected pragmatically to distinguish variables that approximated the sample mean from those showing a relative tendency above or below the mean. Our intention was to support a nuanced interpretation of the relative resource configurations within each cluster. Pairwise comparisons between clusters were conducted using ANOVA with a Games-Howell post hoc test. The Games-Howell test was chosen since it is robust to unequal variances and unequal group sizes, which are common in cluster solutions [49].

Lastly, a gender-based crosstabulation was conducted. Differences in gender distribution between clusters were analysed using a chi-square test. When results were significant (p < 0.05), pairwise chi-square tests were performed to identify which clusters differed in gender representation with post hoc Bonferroni tests to minimise risk of type I error.

### Ethical considerations

The study was approved by the Swedish Ethical Review Authority (Diary number 2024-01088-01), and aligns with the Ethical Review Act, Sections 13–19, which states that guardian consent is not required for persons aged 15–18 [47]. No personally identifiable information was collected, ensuring participant anonymity throughout the data collection process.

## Results

In total, 430 persons aged 15–18 participated in the study, and all 18 national programmes were represented in the sample, with a majority (64%) being enrolled at any of the six higher-education preparatory tracks. Out of the total 430 submitted questionnaires, eleven were deleted due to incomplete answers or observed extreme data, resulting in a total sample size of 419 (girls n = 251 (60%), boys n = 162 (39%), non-binary n = 3 (0.5%) and missing n = 3 (0.5%). Gender comparisons were restricted to participants identifying as girls or boys (n = 413; 251 girls and 162 boys). The three participants identifying as non-binary and the three participants with missing gender information were therefore not included in the gender-comparison analyses.

The distribution of scale scores was examined using the Shapiro–Wilk test together with inspection of skewness and kurtosis. The Shapiro–Wilk test indicated deviations from normality for most variables (p < 0.05), however, this test is highly sensitive in large samples. When skewness and kurtosis values were examined, they were within commonly accepted limits (approximately −1.5 to +2), indicating no meaningful departures from normality [48]. Given the large sample size and the reasonably balanced group sizes, independent samples t-tests were used to compare girls and boys to the total scores of each instrument. Table 2 presents the mean scores for all variables for the total sample and by gender, as well as the p-values from independent t-tests comparing girls and boys. The comparison indicated that there was a significant difference between genders in salutogenic health, PYD self-worth, ego orientation and physical activity level, where boys reported higher levels on all these variables (p-value <0.001 for all four comparisons).

**Table 2:** Descriptive statistics of scores on each variable with gender comparison.

Variable	SHIS	PYD self-worth	PYD pro-social	SMAP	Task	Ego	Physical activity level
	Mean (SD)						
Total (N = 419)	49.99 (11.43)	22.33 (5.71)	16.55 (4.64)	3.68 (0.78)	4.06 (0.71)	3.47 (0.82)	4.90 (1.24)
Girls (n = 251)	46.86 (10.67)	21.04 (4.88)	16.85 (4.38)	3.63 (0.75)	4.02 (0.71)	3.31 (0.81)	4.64 (1.29)
Boys (n = 162)	55.25 (10.57)	24.42 (6.28)	16.17 (4.89)	3.78 (0.81)	4.14 (0.68)	3.73 (0.75)	5.30 (1.03)
p-value^a^	<0.001	<0.001	0.139	0.052	0.45	<0.001	<0.001

^a^independent t-test sign p < 0.05 The overall result on the individual variables demonstrated moderate levels (based on means and possible ranges) of salutogenic health, both PYD domains self-worth and pro-social, and playfulness. Mean score for task orientation was higher than ego orientation and general physical activity level showing relatively limited dispersion, with the majority of the sample reporting to be rather active.

### Hierarchical cluster analysis

The hierarchical cluster analysis suggested a three-cluster solution based on visual inspection of the dendrogram, which was confirmed by the agglomeration schedule.

### K-mean cluster

Based on the hierarchical cluster analysis, a three-cluster solution was selected and subsequently applied in the k-means cluster analysis. The final k-means cluster solution, including the three cluster profiles and cluster comparisons are presented in Table 3.

**Table 3:** Patterns of Z-scores on the various outcome measures for each cluster, along with verbal descriptors and cluster descriptions.

	n	SHIS	PYD self worth	PYD pro-social	SMAP	Task (TEOSQ)	Ego (TEOSQ)	Activity level	Description
**Cluster 1**	108	**Low**	**Low**	**Low**	**Low**	**Low**	**Low**	**Low**	**Low-resource group:** Persons with generally low engagement and limited access to intra- and interpersonal resources supporting agency and resilience.
Mean		-0.633	-0.763	-0.528	-0.816	-0.997	-0.557	-0.753	
SD		0.759	0.571	0.846	1.006	0.740	0.889	1.205	
95% CI		-0.778; -0.488	-0.872; -0.654	-0.690; -0.367	-1.007; -0.624	-1.118; -0.836	-0.073; -0.388	-0.983; -0.523	
**Cluster 2**	116	**High**	**High**	**High**	**High**	**High**	**High**	**High**	**High resource group:** Persons scoring high on all measures indicating high levels of agency and resilience capacities.
Mean		0.817	1.142	0.939	0.687	0.685	0.434	0.432	
SD		0.808	0.873	0.944	0.767	0.616	0.939	0.632	
95% CI		0.668; 0.965	0.981; 1.303	0.765; 1.112	0.546; 0.828	0.572; 0.799	0.261; 0.607	0.316; 0.548	
**Cluster 3**	195	**Medium**	**Low**	**Low**	**Medium**	**High**	**Medium**	**Medium**	**Asymmetric resource group:** Persons with potential internal assets but seems to lack support and secure contexts to explore their intrapersonal assets for agency and resilience.
Mean		-0.085	-0.257	-0.266	0.085	0.255	0.124	0.207	
SD		0.804	0.594	0.691	0.730	0.598	0.857	0.727	
95% CI		-1.199; 0.028	-0.341; -0.173	-0.364; -0.168	-0.018; 0.189	0.171; 0.340	0.003; 0.245	0.105; 0.310	
**Difference**									
C1-C2 ^a)^		<0.001	<0.001	<0.001	<0.001	<0.001	<0.001	<0.001	
C1-C3 ^a)^		<0.001	<0.001	<0.001	<0.001	<0.001	<0.001	<0.001	
C2-C3 ^a)^		<0.001	<0.001	<0.001	<0.001	<0.001	<0.001	<0.001	

Interpretation of Z-scores: High => +0.2; Medium = -0.2 to +0.2; Low = <-0.2

SD = Standard Deviation; CI = Confidence Interval; TEOSQ = Task and Ego Orientation in Sport Questionnaire; SMAP = Short Measure for Adult Playfulness; SHIS = Salutogenic Indicator Scale; PYD = Positive Youth Development

a) ANOVA with a post hoc Games-Howell tests

### Gender comparisons

Gender analyses were restricted to girls and boys due to the low number of non-binary responses. Non-binary responses and missing gender was, thus, excluded from the analysis. (N = 413; 251 girls and 162 boys). Gender distribution differed significantly between clusters (χ² = 19.68, p < 0.001). Within both the low-resource group (75.2% girls) and the asymmetric-resource group (61.7% girls), girls were overrepresented, whereas the high-resource group showed a more even gender distribution (46.1% girls). Pairwise comparisons indicated significant gender differences between the low- and high-resource groups (p < 0.001) and between the high- and asymmetric-resource groups (p < 0.001), while the difference between the low- and asymmetric-resource groups was not significant after Bonferroni correction (p = 0.025).

## Discussion

The descriptive results showed several gender-related differences within the sample. Girls reported lower SHIS scores and marginally reduced self-worth compared with boys, whereas pro-social tendencies remained largely equivalent across groups. The lower subjective rated health among girls is in line with previous research [3,50] as well as boys scoring higher on the domain of self-worth that includes the experience of competence and confidence [21]. The non-significant difference of pro-social tendencies is interesting as previous research in some cases has highlighted gender differences on these aspects [20]. Furthermore, boys exhibited consistently higher scores on physical activity and ego orientation, which is also in line with previous research [28,29]. Moreover, the sample was a rather active group, and similar activity pattern has been reported in a recent Swedish national report [45]. Hence, the descriptive results indicated that the sample is rather representative.

The findings in this study suggest that young persons with seemingly favourable individual resources may nevertheless exhibit vulnerabilities when these resources are considered as interconnected patterns rather than isolated characteristics. This pattern became evident through the identification of three distinct resource groups. The three distinct resource patterns identified were: a low-resource group, a high-resource group, and an asymmetric-resource group. Unlike the first two, the asymmetric group was characterised by medium-to-high levels of intrapersonal resources (SHIS, SMAP, TEOSQ, and physical activity) but comparatively low levels of the relational PYD dimensions of self-worth and prosocial behaviour. These patterns suggest that agency and resilience may be reflected in different configurations of personal and relational resources rather than along a simple continuum from low to high.

The high-resource cluster illustrates how positive experiences and perceived health interact with playful engagement, goal orientation and high physical activity, which is in line with previous research [23,29,51]. An interesting aspect to discuss is whether high levels are always positive. The pro-social dimension of PYD has previously been shown that very high levels of caring and moral aspects can be maladaptive resulting in over-investment in others’ feelings, which can lead to an inability to distinguish between one’s own and others’ emotions, blurring boundaries and causing burnout [52]. In this group however, the self-worth dimension is even more positive indicating that the pro-social behaviours in relation to others seems to come from a secure place indicating a potential ability to distinguish between one’s own and others’ emotions. This contrasts with the low-resource group where PYD self-worth is lower than PYD pro-social, hence the phenomenon of over-investment in other’s feeling might be present in this cluster, and it is worth pinpointing that these dimensions need to be analysed together [40].

The asymmetric group may reflect young persons with a form of hidden potential, indicated by high task orientation and slightly above average levels of playfulness, subjective health, and physical activity. The persons in this group do however score low on PYD self-worth and pro-social. These dimensions include the relational aspects of experienced positive youth development and may indicate challenges related to relational dimensions of positive youth development rather than the lack of intrapersonal resources, something that the Adolescent Well-being Framework emphasise and that the target group has expressed themselves as partly shaping their own health behaviours and well-being [1,53]. This highlights the importance to discuss whether agency and resilience should be separated from connectedness in terms of specific domains within the framework as proposed [1]. Separating domains can aid in theory to fully grasp a definition, however, in application separation can potentially disguise underlying complex patterns. Hence, if only intra-personal resources (SHIS, SMAP, TEOSQ, and physical activity level) had been considered, these persons might have mistakenly been taken for resourceful.

The results of the asymmetric group encourage health promotion initiatives to avoid too individualistic approaches where own behaviours may be accentuated causing young persons’ experience more stress or feeling insufficient. This is also in line with recent research regarding relational definition of resilience in adults and within youth sport [15,16]. Hence it is interesting to see the same pattern in this study´s results. This implies that future research should stay curious about how relationships and connection are part of agency and resilience in health promotion and not too quickly separate these domains with connectedness as this could create adverse effect in health promotion interventions. This could be of special interest to school health services to further explore as the cross-sectional data is taken from persons enrolled within the upper secondary school. Potentially a longitudinal school-intervention related to strengthening relations could benefit the knowledge production about persons’ feelings of agency and resilience.

The result of the relational aspects draws attention to the dynamics that arise between persons and their contexts. These interactions could potentially be influenced by power imbalances. Particularly when considering young persons in school settings where a power imbalance can occur due to the adult being seen as the expert in this environment and the adults’ behaviours can both encourage and hinder young persons’ health behaviours [53,54]. It is noteworthy that, within the asymmetric-resource group, the lower ratings on PYD self-worth and PYD pro-social may be shaped by a perceived power imbalance in relationships and/or contexts in which the young persons are situated. Such imbalances may contribute to experiences of reduced support, diminished self-confidence, and constrained access to their full potential and rights. This potential power imbalance is of interest for future research as the PYD framework challenges the perspective that young persons are problems to be solved by adults or ‘experts’ [13].

Recognising the complexity and relational nature of the identified resource patterns also highlights the relevance of person-centred approaches in health promotion. By positioning young persons as experts on their own lived experiences, person-centredness offers one way of addressing the power imbalances. A person-centred approach positions the young persons as the experts of their lived experience, foregrounding their values, inner emotions, preferences, and motivations [55]. The approach aspires to establish a shared partnership for health, creating space for all to express their authentic selves and create trust. Such conditions lay the groundwork for the development of relationships that are both safe and secure for young persons to realise their potential and experience well-being [55]. Furthermore, a person-centred approach may inform young persons of the power of relationships, both for their own well-being and also in relation to others’ well-being. Person-centredness as a strategy has been proven to be successful in therapeutic sessions for young persons where they describe a sense of secure space to be themselves as the power imbalance was reduced [56]. Hence, exploring the person-centredness framework in relation to promoting young persons’ well-being is of further interest.

In the final cluster solution, girls were also more represented in the low-resource group and the asymmetric-resource group, hence informs us of a wide diversity within this subgroup. Furthermore, the response pattern on the physical activity item indicates that the sample seems to be physically active in general, but despite this there was still a significant difference in physical activity level between genders. Boys were significantly more physically active than girls. This is in line with previous research where girls in this age group tend to leave physical activities and sports earlier than boys and where too much focus on competition is one reason [28,57,58]. The interesting part in the results of this study is that girls and boys did not significantly differ in task orientation, which has been associated with a higher level of sport participation over time [29]. However, boys did score higher on ego orientation, and this combination can be more in line with how sports in a Nordic modern society are developing, where the former participation-oriented sports environments are being replaced with more professional and competitive environments [59]. It is not possible to say whether most of the very active persons in this sample are part of an organised sport or physical activity, but it is worth highlighting that the physical activity level of the asymmetric cluster is on the borderline to “high” and with a “medium” level of subjective health. Hence, the results signal that future research should widen the perspective of how physical activity and potential sport participation relate to well-being and health-related outcomes and focus on processes that might influence the relational and gender perspective. Gadsby and Wilding [60] refer to this as a broader perspective of system thinking within health promotion. In their distinction, a narrower systematic perspective (which assumes that the world consists of clearly defined problems that can be solved by identifying the “right” intervention) is contrasted with the broader systemic perspective, where the world is viewed as problematic, complex and shaped by multiple coexisting truths. This broader perspective shifts attention away from a linear, individual-level approach, such as whether a person is physically active or not, towards creating space for learning, questioning and understanding the relationships and structures surrounding the person. Such an approach enables a deeper understanding of both the gender differences in the results and the diversity among the girls in the sample.

To summarise, the results are in line with the overall Adolescent’s Well-being Framework that emphasises both personal resources and supportive relationships to thrive and experience well-being [1,4]. However, the results support that the agency and resilience domain of the framework should be considered alongside the connectedness domain as the relational resources within PYD separated the clusters. The findings illustrate the importance of understanding young persons’ well-being through interconnected patterns of resources rather than isolated characteristics.

### Strengths and weaknesses

Cluster analysis is an exploratory and descriptive method, and the resulting cluster solution may be influenced by methodological choices such as clustering procedure, distance measures, and the number of clusters selected. Alternative clustering methods or different selections of included variables may have resulted in somewhat different cluster solutions. Therefore, the identified patterns should not be interpreted as fixed or universally generalizable categories, but rather as context-bound descriptions that may contribute to reflection and future hypothesis generation. The cluster analysis was performed mainly on subjective ordinal raw data (except PYD self-worth and pro-social). Despite transforming to Z-score the underlying ordinal data might affect the interpretations. Validity and reliability can always be discussed in relation to the individual measurements but being frequently used with acceptable validity in research all instruments were motivated to use in the study. However, increased availability of linear measurements for the different latent variables would be desirable. Moreover, more accurate and feasible approaches could be used to measure physical activity level such as multi-options instruments, or other objective measures, e.g., accelerometers and activity logs to provide a better understanding and accurate assessment. Furthermore, researchers may use observational methods for specific situations, also it may not fully explain or fit on overall activity level.

There was a slight uneven distribution between genders where a majority of the persons in the study were girls. The persons in the sample were very active, and this might have affected the results. However, the results are in line with a previous report in Sweden [45]. It could be argued that physical activity should be measured in a non-self-reported way to avoid potentially different interpretations of the response categories.

As the persons in the sample is in their development phase further longitudinal research is needed to gain understanding if the cluster patterns are part of or susceptible to natural change over time due to maturation. The results describe patterns within the sample that can inform future qualitative research in terms of exploring agency and resilience with focus on PYD relational processes in the age group. As previous literature has described agency and resilience are complex concepts, and more aspects needs to be explored to gain further understanding of how it can relate to well-being for persons aged 15–18 [2,16]. This could also shed a light on how cultural aspects and socioeconomic factors might influence agency and resilience [61], as well as addressing that the age 15–18 is a time where young persons might search and questioning their belonging and might move away from home to attend different upper secondary schools.

## Conclusions

In conclusion, the clusters that appeared in the study illustrate the complexity of agency and resilience, and that it is not only a matter of high, medium, or low levels of a particular intra- or interpersonal resources. Rather, there is a need for a continued discussion in which agency and resilience are understood in conjunction to relational aspects and resources. In other words, further discussion within for example school health promotion is needed to widen the perspectives of health beyond single factors or domains of health. Most importantly, these discussions should continue to explore the relational aspects and resources, as well as further exploring the diversity observed among the girls. Future research may benefit from theoretical perspectives such as person-centredness, and a wider perspective of system thinking to gain a deeper understanding of gender differences. Such knowledge can inspire and aid school health services to innovative school health promotion initiatives.
