# Supplementary material for: Agency and resilience among young persons in Sweden: Patterns of interrelated resources within the WHO Adolescent Well-being Framework
Source: PLoS One. 2026 Oct 30;21(10):e0360518. doi: 10.1371/journal.pone.0360518 (PMC13722179; doi:10.1371/journal.pone.0360518)
Supplement: Supplementary file 2 [file pone.0360518.s001.docx]

**Manuscript Title: Emergent patterns of agency and resilience among Swedish persons aged 15-18: A school health promotion perspective**

**Manuscript Number: PONE-D-26-05672**

**Reviewer:** Dr. Qurat ul Ain

**1. Summary of the Manuscript**

This research focused on different patterns of behaviours (agency and resilience ) among adolescent ages between 15-18. The purpose of this research is to explore and measure different aspects of children’s behaviours towards positive physical and mental health promotion, specifically salutogenic health, PYD , playfulness, task & ego-orientation and physical activity that affect them in their later life.

**2. Major Assessment**

Researchers used appropriate and well structured research methods to measure the patterns of behaviour among adolescents. The use of a cross-sectional research design is suitable to identify the relationship within the data-set for this manuscript. Despite the fact, cross-sectional research design has no temporal sequence, and limits the ability to present causal conclusions. However, researchers used self-reported valid and reliable instruments which enhanced the credibility of research findings; though the self- reported instrument may create response bias. Furthermore, the use of a single item may not completely measure the complexity of the variable.

The use of cluster analysis within the multiple interrelated variables was the basic strength of the study’s aim. Other statistical methods such as chi-square were accurate and relevant to examine gender differences within clusters. Despite the strengths, according to the study nature, researchers may but not limited to acknowledging that cluster analysis can be sensitive to methodology choice and it may affect the results generalizability.

The present research study is useful and acceptable and good for understanding patterns. For measuring physical activity with a single item is a limitation. For more accurate and feasible approaches such as multi-options instruments, other objective measures e.g accelerometers and activity logs may explore a better understanding and accurate assessment. Furthermore, researchers may use observational methods for specific situations, also it may not fully explain or fit on overall activity level.

**3. Major Comments**

Overall, the use of research methodology is relevant but the finding of the study should be defined well by considering the limitations and nature of the research design.

**4. Minor Comments**

- Grammatical errors required attention in the conclusion part to be corrected on (p.20). Line 515-516 “Future research may benefit from theoretical perspective such as” (plural) perspectives.
- Conclusion section (p.20) line 514 “ have a continued curiosity” is acceptable. However, more neutral and accurate way to explain could be “maintain continued curiosity or continue to explore”
- Conclusion section(p.20) line 516”the discussion should continue within school health promotion “ to “further discussion within school health promotion is needed”

Discussion section (p.18). Line 465” a wider perspective of system thinking, a better way to write or explain “a system thinking perspective.

- By defining “adolescents”ages between 15-18 (Early or middle Phase) Authors can include reference, World Health Organization for concise and research objective clarity if applicable.

**5. Recommendation**

**Recommended Revision**

The study highlights the behavior patterns in adolescents ages (15-18) that show how positive characteristics combine to shape their resilience and self-development within gender differences. The manuscript is well written and well structured according to the PLOS standards. However, the study needs some clarifications on methodology to cover the research methods limitations and needed some revisions in conclusion. It is also recommended that future research should use more comprehensive and objective measurement tools and adopt longitudinal designs to strengthen the validity and depth of the findings.

I recommend this work should be published with minor revisions and improvements.
