# Supplementary material for: Agency and resilience among young persons in Sweden: Patterns of interrelated resources within the WHO Adolescent Well-being Framework
Source: PLoS One. 2026 Oct 30;21(10):e0360518. doi: 10.1371/journal.pone.0360518 (PMC13722179; doi:10.1371/journal.pone.0360518)
Supplement: Supplementary file 3 [file pone.0360518.s004.docx]

Thank you for the opportunity to submit a revised version of our manuscript (ID: PONE-D-26-05672), originally entitled *Emergent patterns of agency and resilience among Swedish persons aged 15-18: A school health promotion perspective.* Note that after the valuable and significant feedback we received from the reviewers we have decided to re-name the article to *Agency and resilience among young persons in Sweden: Patterns of interrelated resources within the Adolescent Well-being Framework.*

We sincerely appreciate the valuable feedback and are grateful to the reviewers for their insightful and constructive comments. Based on these suggestions, we have made corresponding revisions throughout the manuscript.

We look forward to your response and remain happy to address further questions or comments.

Please find below our point-by-point response to the journal requirements and reviewer’s comments and concerns.

**Editor comments:**

**Comment 1**: Please ensure that your manuscript meets PLOS ONE's style requirements, including those for file naming. The PLOS ONE style templates can be found at

<https://journals.plos.org/plosone/s/file?id=wjVg/PLOSOne_formatting_sample_main_body.pdf> and

[https://journals.plos.org/plosone/s/file?id=ba62/PLOSOne_formatting_sample_title_authors_affiliations.pdf](https://track.editorialmanager.com/CL0/https:%2F%2Fjournals.plos.org%2Fplosone%2Fs%2Ffile%3Fid=ba62%2FPLOSOne_formatting_sample_title_authors_affiliations.pdf/1/010f019e396dbb21-8c658193-0bd9-4c8e-af82-b679e9c138c5-000000/quu-7T4Z8Ta7rAf7tTYgsEJiVoPgfw3BTpCtJDtxVeE=258)

*Response:* Many thanks for highlighting the guidelines and the title page and headings have been revised accordingly and the supporting files have been changed to “other”.

**Comment 2:** You indicated that you had ethical approval for your study. In your Methods section, please ensure you have also stated whether you obtained consent from parents or guardians of the minors included in the study or whether the research ethics committee or IRB specifically waived the need for their consent.

*Response:*

We appreciate that the editor pinpointing this and the text has been updated in the Method section in relation to guardians’ consent as follows:

“, and according to the Swedish Ethical Review Act section 13-19, guardians’ consent is not required for participants aged 15-18. No personal information was collected; hence, anonymity was maintained during and after the data collection.”

**Comment 3**: Thank you for stating the following financial disclosure:

“Kristianstad University”

Please state what role the funders took in the study.  If the funders had no role, please state: "The funders had no role in study design, data collection and analysis, decision to publish, or preparation of the manuscript."

If this statement is not correct you must amend it as needed.

Please include this amended Role of Funder statement in your cover letter; we will change the online submission form on your behalf.

*Response:* "The funders had no role in study design, data collection and analysis, decision to publish, or preparation of the manuscript." We are grateful that this will be changed in the online submission form on our behalf.

**Comment 4**: We note that you have indicated that there are restrictions to data sharing for this study. PLOS only allows data to be available upon request if there are legal or ethical restrictions on sharing data publicly. For more information on unacceptable data access restrictions, please see <http://journals.plos.org/plosone/s/data-availability#loc-unacceptable-data-access-restrictions>.

Before we proceed with your manuscript, please address the following prompts:

a) If there are ethical or legal restrictions on sharing a de-identified data set, please explain them in detail (e.g., data contain potentially identifying or sensitive patient information, data are owned by a third-party organization, etc.) and who has imposed them (e.g., a Research Ethics Committee or Institutional Review Board, etc.). Please also provide contact information for a data access committee, ethics committee, or other institutional body to which data requests may be sent.

*Response:* We appreciate the editor noticing these restrictions. There is an ethical restriction as the ethical approval for the PhD project includes not sharing data with third parties and can only be used by any member of the research project team.

Original text from the ethical application approved by the Swedish Ethical Review Committee:

***6.4 Hur kommer insamlade data att hanteras och förvaras?***

*Eftersom datainsamling sker anonymt kommer all data i de båda studierna att vara anonym. Det kommer alltså inte att finnas några kodlistor eller kodnycklar. Det insamlade datamaterialet sparas enligt Högskolan Kristianstads regler för hantering av forskningsdata. Datamaterialet lagras enbart på godkänd server för forskningsdata. Det är enbart medverkande forskare i projektet som kommer att ha tillgång till det insamlade datamaterialet.*

Translated original text:

***6.4 How will the collected data be managed and stored?***
*Since the data collection is conducted anonymously, all data in both studies will be anonymous. This means that no code lists or code keys will exist. The collected data will be stored in accordance with Kristianstad University’s regulations for the management of research data. The data will be stored only on an approved server for research data. Only the researchers participating in the project will have access to the collected data.*

The full name of the body imposing the restrictions upon your dataset (ethics committee, institution, data access committee is The Swedish Ethical Review Authority. Data access requests can be sent is Swedish Ethical Review Authority: [registrator@etikprovning.se](mailto:registrator@etikprovning.se). The diary number for the research project is: 2024-01088-01.

The data set and its metadata has been uploaded via Swedish National Data Service (SND) and has a reserved DOI https://doi.org/10.5878/1dn8-qj65. The metadata will be fully available. Many thanks for updating the Data Availability statement on our behalf.

**Comment 5:** Please include captions for your Supporting Information files at the end of your manuscript, and update any in-text citations to match accordingly. Please see our Supporting Information guidelines for more information: [http://journals.plos.org/plosone/s/supporting-information](http://track.editorialmanager.com/CL0/http:%2F%2Fjournals.plos.org%2Fplosone%2Fs%2Fsupporting-information/1/010f019e396dbb21-8c658193-0bd9-4c8e-af82-b679e9c138c5-000000/I1W_jrb-JG7wSUKfXnlgU5R0jwB4DCHENf_ylrf6RUo=258).

*Response:* Many thanks for highlighting this as we notice that these files should have been added as “other” and not “Supporting Information”, this has been changed in the new submission and there are no Supporting Information Files.

**Comment 6**: Thank you for stating the following financial disclosure:

“Kristianstad University”

We note that one or more of the authors is affiliated with the funding organization, indicating the funder may have had some role in the design, data collection, analysis or preparation of your manuscript for publication; in other words, the funder played an indirect role through the participation of the co-authors. If the funding organization did not play a role in the study design, data collection and analysis, decision to publish, or preparation of the manuscript and only provided financial support in the form of authors' salaries and/or research materials, please do the following:

1. Review your statements relating to the author contributions, and ensure you have specifically and accurately indicated the role(s) that these authors had in your study. These amendments should be made in the online form.

*Response:* We thank the editor for highlighting this and the roles have been accurately indicated.

2. Confirm in your cover letter that you agree with the following statement, and we will change the online submission form on your behalf:

“The funder provided support in the form of salaries for authors [JB, AW, ÅB, PNL and MH], but did not have any additional role in the study design, data collection and analysis, decision to publish, or preparation of the manuscript. The specific roles of these authors are articulated in the ‘author contributions’ section.

*Response:* We hereby confirm that the author contributions are right and the above statement is true and many thanks for changing the online submission form on our behalf.

**Reviewer #1**

**Comment 1:** A valid and sound research paper, however the title might be reword as not to include the age-specific of the respondents.

*Response:* We thank the reviewer for this valuable suggestion relating to the title. After consideration of all significant input on the manuscript we have decided to change the title to: “Agency and resilience among young persons in Sweden: Patterns of interrelated resources within the Adolescent well-being framework.”

**Comment 2:** More depth needed to strengthen the overall research framework.

*Response:* We appreciate the reviewer's thoughtful feedback, and the logic structure of the background has now been re-structured to emphasise the WHO Adolescent well-being framework. The new structure of the background is:

- WHO adolescent well-being framework
- Highlighting the importance of the agency and resilience domain within the framework
- The research gap within the domain
- How the domain can be operationalised and why the chosen constructs are relevant
- The contribution of the present study
- The aim of the study

**Comment 3:** Previous studies may contrast the current findings & need to address them accordingly.

*Response:* Thank you for highlighting the need to contrast the findings. By revising the background and adding references in relation to gender differences we hope to have strengthened this perspective of the study. The reference Sarkar & Page (2022) has also been added in the discussion to address the fact that the study show encourage the approach that agency and resilience should not be studied in isolation from connectedness. Sarkar & Page (2022) highlight that resilience is more complex than being an individualistic trait and the environment and support needs to be addressed more in research.

 -----------------

**Reviewer #2:** 

**Comment 1:** Researchers used appropriate and well structured research methods to measure the patterns of behaviour among adolescents. The use of a cross-sectional research design is suitable to identify the relationship within the data-set for this manuscript. Despite the fact, cross-sectional research design has no temporal sequence, and limits the ability to present causal conclusions. However, researchers used self-reported valid and reliable instruments which enhanced the credibility of research findings; though the self- reported instrument may create response bias. Furthermore, the use of a single item may not completely measure the complexity of the variable. The use of cluster analysis within the multiple interrelated variables was the basic strength of the study’s aim. Other statistical methods such as chi-square were accurate and relevant to examine gender differences within clusters. Despite the strengths, according to the study nature, researchers may but not limited to acknowledging that cluster analysis can be sensitive to methodology choice and it may affect the results generalizability.

*Response:* We thank the reviewer for this important comment. We have clarified the exploratory nature of the cluster analysis and emphasized that the identified patterns should not be interpreted as fixed or universally generalizable. We also added information that alternative cluster solutions were explored, and that a four-cluster solution was rejected due to small cluster sizes and limited interpretability, supporting the final three-cluster solution.

In the *Statistical analysis* section, the following text was added:

“Alternative cluster solutions were explored during the analytical process. A four-cluster solution was considered but rejected due to small cluster sizes and limited interpretability, supporting the robustness and interpretability of the final three-cluster solution.”

In the *Strengths and Weaknesses* section, the following paragraph was revised:

“Cluster analysis is an exploratory and descriptive method, and the resulting cluster solution may be influenced by methodological choices such as clustering procedure, distance measures, and the number of clusters selected. Therefore, the identified patterns should not be interpreted as fixed or universally generalizable categories, but rather as context-bound descriptions that may contribute to reflection and future hypothesis generation.”

**Comment 2:** The present research study is useful and acceptable and good for understanding patterns. For measuring physical activity with a single item is a limitation. For more accurate and feasible approaches such as multi-options instruments, other objective measures e.g; accelerometers and activity logs may explore a better understanding and accurate assessment. Furthermore, researchers may use observational methods for specific situations, also it may not fully explain or fit on overall activity level.

*Response:* We appreciate this important feedback in relation to our one-item subjective rating in relation to physical activity. The Strengths and weaknesses section has been updated accordingly to reviewers comment:

“Moreover, more accurate and feasible approaches could be used to measure physical activity level such as multi-options instruments, or other objective measures e.g; accelerometers and activity logs to provide a better understanding and accurate assessment. Furthermore, researchers may use observational methods for specific situations, also it may not fully explain or fit on overall activity level.”

**Comment 3:** Overall, the use of research methodology is relevant but the finding of the study should be defined well by considering the limitations and nature of the research design.

*Response:* Many thanks for this overall comment and please see above responses where we have addressed these limitations accordingly.

**Comment 4:** Grammatical errors required attention in the conclusion part to be corrected on (p.20). Line 515-516 “Future research may benefit from theoretical perspective such as” (plural) perspectives.

*Response:* Thank you for noticing this grammatic error. It has been changed accordingly and is now written in plural.

**Comment 5:** Conclusion section (p.20) line 514 “ have a continued curiosity” is acceptable. However, more neutral and accurate way to explain could be “maintain continued curiosity or continue to explore”

*Response:* We are grateful for this helpful observation and we have changed accordingly and the text is now phrased as “continue to explore”.

**Comment 6:** Conclusion section(p.20) line 516”the discussion should continue within school health promotion “ to “further discussion within school health promotion is needed”

*Response:* Thank you for suggesting this re-phrasing and the text has been updated accordingly.

**Comment 7:** Discussion section (p.18). Line 465” a wider perspective of system thinking, a better way to write or explain “a system thinking perspective.

*Response:* We sincerely thank the reviewer for this comment suggesting this re-phrasing. However, this phrasing is linked to the wording of the reference Gadsby and Wilding (2024), hence instead the texts is now consistently using the term broader instead that is also used in the upcoming sentence where the term is explained more explicitly.

**Comment 8:** By defining “adolescents” ages between 15-18 (Early or middle Phase) Authors can include reference, World Health Organization for concise and research objective clarity if applicable.

*Response:* We appreciate the reviewer's excellent suggestion and as the title has changed and the WHO adolescent well-being framework is more central in the study, the WHO reference in relation to adolescence has now been added in the first section of the Background:

“As researcher emphasise the importance of the developmental years, a specific definition addressing well-being for persons aged 10-19 has been proposed by World Health Organisation (WHO) for guiding international policy development”

**Comment 9:** The study highlights the behavior patterns in adolescents ages (15-18) that show how positive characteristics combine to shape their resilience and self-development within gender differences. The manuscript is well written and well structured according to the PLOS standards. However, the study needs some clarifications on methodology to cover the research methods limitations and needed some revisions in conclusion. It is also recommended that future research should use more comprehensive and objective measurement tools and adopt longitudinal designs to strengthen the validity and depth of the findings.
I recommend this work should be published with minor revisions and improvements.

*Response:* We greatly appreciate the reviewer's careful reading of our manuscript*.* We hope that the adjustments to the manuscripts following the valuable input has highlighted the clarifications in relation to method and the conclusions based on the method limitations.

-----------------------------

**Reviewer #3:** 

**Comment 1-4:** the literature review is thorough and covers the necessary literature. However, I felt it could be more tightly focused to guide the reader to the paper’s goals. For example, the authors’ note that the agency and resilience domain might be understudied in terms of health because of a different focus in psychological literature seems unrelated to the goals of the paper. Looking for material like that and modifying or removing it could help sharpen the argument and keep the reader attached to the main goals of the paper.

--along these lines, the literature review would benefit from a more animating reason for looking at agency and resilience than that a systematic review divided literature into segments that named an agency and resilience segment. Youth agency in a world structured for extraction is a fascinating and important topic, and the authors could make it a more powerful framework to support the inquiries here.

--the literature review could use more clarity as to why the six domains are important and why they are specifically examined here. This may be an issue of wanting to broaden appeal a little to a interdisciplinary audience; as an example, I am intensely interested in the authors’ questions about agency and resilience, but I am not familiar with Ross and colleagues, who appear to be in public health. A little more orientation about these domains and why the current authors choose them would help extend the usefulness of the current paper to a broader audience.

--as an example of the above, it is unclear to me what playfulness has to do with PYD perspectives specifically. Perhaps another way to think of this is that the overall framework of the paper claims to be about agency and resilience, but much of the literature review is focused on much more granular ideas without connecting them explicitly to agency and resilience.

*Response:* We are grateful for the reviewer's constructive feedback, which has helped improve the manuscript. The above four comments made us re-consider the structure of the Background. The text has been revised to further highlight that the study is grounded in the WHO Adolescent Well-Being Framework and its agency and resilience domain. The new structure is accordingly:

- WHO adolescent well-being framework
- Highlighting the importance of the agency and resilience domain within the framework
- The research gap within the domain
- How the domain can be operationalised and why the chosen constructs are relevant
- The contribution of the present study
- The aim of the study

**Comment 5:** along the lines of focusing the literature review, gender differences are an important part of the findings, but they are not well set up in the literature review. It might be useful to think about the article as a piece of paper that could be folded on the methods section; the literature review pieces should match the discussion pieces they “touch” when the paper is folded in order to craft the clearest argument and then present the clearest evidence for that argument.

*Response:* We appreciate this constructive comment. The first paragraph of the Background section is now updated with a gender perspective on self-reported health in relation to adolescence and is based upon Health Behaviour in School-aged children study report from 2021/2022.

**Comment 6:** it is unclear to me from the sample section what the N actually is and any exclusion criteria or issues of missing data. This may be a disciplinary question, given later information under “data collection,” but I found this organization confusing. That said, I am still confused about the N, or perhaps the issue is Ns, since there may be data from a small number of cognitive interviews and a larger number of survey respondents? The fact that I’m not sure is a sign of an important problem, though one that can likely be easily resolved through writing revisions rather than any data issue.

*Response:* We appreciate the reviewer's comment for highlighting this experienced confusion. The use of N follows praxis in statistical report where N = total number of sample and n=number of persons/cases within a subgroup within the sample.

**Comment 7:** some sample questions from the PYD-VSF would be useful; the SHIS section is a good example of doing this well. This applies to the other included constructs as well.

--if space is an issue, I personally feel as though the references to validation of scales could be trimmed to saying they had been validated and a citation. Then that space could be used for things like sample questions from the scale.

*Response:* Many thanks for valuable feedback on presenting the instruments. In relation to PYD-VSF the five different components have varied items and we have re-phrased the presentation to focus more on the instrument rather than than the model and to summarise what the items represent within each “C”:

“The instrument is based on the The 5C model within PYD framework (8). The model identifies the five core components that builds up PYD-VSF: Competence, Confidence, Connection, Character, and Caring (40). Items related to *Competence* refers to a positive evaluation of one's performance in specific domains, including social, academic, cognitive, health, and vocational areas. *Confidence* items denotes an internal sense of overall self-worth and perceived ability. *Connection* items captures the presence of positive, bidirectional bonds with people and institutions, such as peers, family, school, and community, emphasizing the mutual nature of these relationships. *Character* items is defined relate toas respect for societal and cultural norms, adherence to moral standards, a well-developed sense of right and wrong, and integrity*. Caring* items involves both the experience of being cared for and caring for others and is associated with feelings of sympathy and empathy.”

The shorter instruments of SMAP and TEOSQ have also been updated with example of items.

**Comment 8:** gender is a key component of the analysis, but there is no description of how gender was measured.

*Response:* We are grateful for this helpful observation and this has been updated in the text under Instrument section:

“gender identification (girl, boy, non-binary, other or do not want to answer),”

**Comment 9:** the authors state that they imposed levels of low, medium and high to cluster profiles because they “considered [this threshold] appropriate and meaningful.” Why? Greater attention to arguing for these choices will help build confidence in findings.

*Response:* We thank the reviewer for this valuable comment. We have clarified that the labels “low,” “medium,” and “high” were used as descriptive aids to facilitate interpretation of relative differences between cluster profiles rather than as normative cut-off values. We further clarified that the thresholds were based on standardized Z-scores, where values close to zero represent average levels within the sample.

In the *Statistical analysis* section, the following paragraph was revised:

“We applied Z-score thresholds of ±0.2 to interpret the cluster profiles in terms of ‘low,’ ‘medium,’ and ‘high’ levels across the included variables. These thresholds were used as heuristic and descriptive aids to facilitate interpretation of relative differences between cluster profiles rather than as normative or clinical cut-off values. Values close to zero were interpreted as representing average levels within the sample, while deviations above or below ±0.2 were considered to indicate meaningful tendencies in the cluster patterns. Our intention was to support a nuanced interpretation of the relative resource configurations within each

**Comment 10:** a final N (I assume!) finally appears in the results section, which is a difficult placement. In addition, it would be helpful to know why, out of a potential 375,000 students we are looking at 430. This is not necessarily fatal; it is just important to know how these sampling details might affect interpretation of results.

*Response:* We appreciate the reviewer bringing this issue to our attention. The N is as responded to in a previous comment the praxis of reporting total sample size short, hence part of the result where we report sample size and the size of the subgroups (such as gender or clusters).

The data collection procedure has been updated to clarify that the schools were purposefully selected and that they were informed about the study and that the distribution of the link and QR code was optional for the schools, hence a large nonresponse could be expected as schools could have chosen not to distribute the link and QR code. We hope this clarifies the procedure and the sample size.

**Comment 11:** it is unclear to me what items represent interpersonal resources. This is really important since so many of the most interesting findings are tied to this concept. Similarly, it is unclear where physical activity was measured and into what domain it would ft.

*Response:* We thank the reviewer for raising this important point. In relation to interpersonal resources in the discussion this has been further explained by adding the relevant instruments related to this:

“If only intra-personal resources (SHIS, SMAP, TEOSQ, and physical activity level) had been considered, these persons might have mistakenly been taken for resourceful. Separating domains can aid in theory to fully grasp a definition, however, in application separation can potentially disguise underlying complex patterns.”

Physical activity has been further explained in the updated Background section to be related to both agency and resilience. Furthermore, the Method section has described that it was measured using a subjective rating single item that has been used in surveys with young persons in Sweden before. Also we have added a comment under Strength and weaknesses section to point out the limitation of subjective rating of physical activity and suggested other methods for future research:

“Moreover, more accurate and feasible approaches could be used to measure physical activity level such as multi-options instruments, or other objective measures e.g; accelerometers and activity logs to provide a better understanding and accurate assessment. Furthermore, researchers may use observational methods for specific situations, also it may not fully explain or fit on overall activity level.”

**Comment 12:** I really appreciated the authors’ discussion about whether high levels are always positive.

*Response:* We sincerely thank the reviewer for this kind comment!

**Comment 13:** there is emphasis on school-based interventions in both the literature review and discussion, but no measures or tests of such things. I wonder if instead this could be introduced in the discussion as a potential intervention that might be useful given the findings.

*Response:* We greatly appreciate the reviewer's careful reading of our manuscript and this valuable influenced our decision to re-structure the Background and removed the school-based approach in the background and is now introduced as part of the discussion:

“This could be of special interest to school health services to further explore as the cross-sectional data is taken from persons enrolled within the upper secondary school. Potentially a longitudinal school-intervention related to strengthening relations could benefit the persons feeling of agency and resilience. “

The school perspective is then maintained when discussing power imbalances and future research as well. Furthemore, this comment made us reflect upon the title and retitled the manuscript to *Agency and resilience among young persons in Sweden: Patterns of interrelated resources within the Adolescent well-being framework.*
