# Supplementary material for: Agency and resilience among young persons in Sweden: Patterns of interrelated resources within the WHO Adolescent Well-being Framework
Source: PLoS One. 2026 Oct 30;21(10):e0360518. doi: 10.1371/journal.pone.0360518 (PMC13722179; doi:10.1371/journal.pone.0360518)
Supplement: Supplementary file 4 [file pone.0360518.s005.docx]

Thank you for the opportunity to submit a revised version of our manuscript (ID: PONE-D-26-05672) *Agency and resilience among young persons in Sweden: Patterns of interrelated resources within the Adolescent Well-being Framework.*

We sincerely appreciate the valuable feedback and are grateful to the reviewers for their insightful and constructive comments. Based on these suggestions, we have made corresponding revisions throughout the manuscript (page and line refers to the document with track changes):

We look forward to your response and remain happy to address further questions or comments.

Please find below our point-by-point response to the journal requirements and reviewer’s comments and concerns.

-----------------------------

**Reviewer #3:** 

**Comment 1:** I found the abstract to be a little difficult to follow and wondered if the paper overall (and searches for literature) might benefit from focusing more on the overall takeaways rather than some analytical details that might be better saved for the text where there is space to more thoroughly explain them.

*Response:* Thank you for this helpful comment. We agree that the main message of the study was not sufficiently prominent and that some analytical detail obscured the overall findings, particularly in the abstract. We have therefore revised the abstract to place greater emphasis on the main findings and their implications. We have also revised relevant sections throughout the manuscript to improve the consistency and clarity of this message. In particular, we now emphasise that the findings illustrate how personal and relational resources form interconnected patterns that may not be apparent when individual resources are considered separately. The corresponding revisions are provided below.

**Background, page 8, line 171-173:**

If agency and resilience emerge through constellations of personal and relational resources rather than isolated factors, examining how these resources coexist may contribute to a more nuanced understanding of the domain.

**Discussion, page 21, line 439-454:**

The findings in this study suggest that young persons with seemingly favourable individual resources may nevertheless exhibit vulnerabilities when these resources are considered as interconnected patterns rather than isolated characteristics. This pattern became evident through the identification of three distinct resource groups. Three distinct resource patterns identified were: a low-resource group, a high-resource group, and an asymmetric-resource group. Unlike the first two, the asymmetric group was characterised by medium-to-high levels of intrapersonal resources (SHIS, SMAP, TEOSQ, and physical activity) but comparatively low levels of the relational PYD dimensions of self-worth and prosocial behaviour. These patterns suggest that agency and resilience may be reflected in different configurations of personal and relational resources rather than along a simple continuum from low to high.

**Discussion, page 22, line 483-487:**

Separating domains can aid in theory to fully grasp a definition, however, in application separation can potentially disguise underlying complex patterns. Hence, if only intra-personal resources (SHIS, SMAP, TEOSQ, and physical activity level) had been considered, these persons might have mistakenly been taken for resourceful.

**Discussion, page 24, line 515-518:**

Recognising the complexity and relational nature of the identified resource patterns also highlights the relevance of person-centred approaches in health promotion. By positioning young persons as experts on their own lived experiences, person-centredness offers one way of addressing the power imbalances.

**Discussion, page 26, line 562-564:**

The findings illustrate the importance of understanding young persons' well-being through interconnected patterns of resources rather than isolated characteristics.

**Comment 2:** how were the 6 non-binary students or those who did not report gender but were still included in the sample treated in the gender comparison? In practical terms, were they excluded, even though they are not listed as excluded?

*Response*: Thank you for identifying this lack of clarity. The reviewer is correct that six participants were not included in the gender comparisons: three participants identified as non-binary and gender information was missing for three participants. These participants remained part of the overall study sample but were excluded only from analyses comparing gender groups. We have therefore clarified this in the manuscript as follows

**Results, page 17, line 358-361**:

“Gender comparisons were restricted to participants identifying as girls or boys (n = 413; 251 girls and 162 boys). The three participants identifying as non-binary and the three participants with missing gender information were therefore not included in the gender-comparison analyses.”

**Results, page 20, line 394-395**:

“Gender analyses were restricted to girls and boys due to the low number of non-binary responses. Non-binary responses and missing gender was, thus, excluded from the analysis”
